# Impact of Providing a Tape Measure on the Provision of Lung-protective Ventilation

**DOI:** 10.5811/westjem.2020.10.49104

**Published:** 2021-01-11

**Authors:** Crystal M. Ives Tallman, Carrie E. Harvey, Stephanie L. Laurinec, Amanda C. Melvin, Kimberly A. Fecteau, James A. Cranford, Nathan L. Haas, Benjamin S. Bassin

**Affiliations:** * University of Michigan Medical School, Department of Emergency Medicine, Ann Arbor, Michigan; † University of Michigan Medical School, Department of Anesthesiology/Critical Care, Ann Arbor, Michigan; ‡ University of Michigan Medical School, Division of Emergency Critical Care, Ann Arbor, Michigan; § University of Michigan Medical School, Department of Respiratory Care, Ann Arbor, Michigan; ¶ University of Michigan, Center for Integrative Research in Critical Care, Ann Arbor, Michigan

## Abstract

**Introduction:**

Emergency department (ED) patients are frequently ventilated with excessively large tidal volumes for predicted body weight based on height, which has been linked to poorer patient outcomes. We hypothesized that supplying tape measures to respiratory therapists (RT) would improve measurement of actual patient height and adherence to a lung-protective ventilation strategy in an ED-intensive care unit (ICU) environment.

**Methods:**

On January 14, 2019, as part of a ventilator-associated pneumonia prevention bundle in our ED-based ICU, we began providing RTs with tape measures and created a best practice advisory reminding them to record patient height. We then retrospectively collected data on patient height and tidal volumes before and after the intervention.

**Results:**

We evaluated 51,404 tidal volume measurements in 1,826 patients over the 4 year study period; of these patients, 1,579 (86.5%) were pre-intervention and 247 (13.5%) were post-intervention. The intervention was associated with a odds of the patient’s height being measured were 10 times higher post-intervention (25.1% vs 3.2%, P <0.05). After the bundle was initiated, we observed a significantly higher percentage of patients ventilated with mean tidal volumes less than 8 cubic centimeters per kilogram (93.9% vs 84.5% P < 0.05).

**Conclusion:**

Patients in an ED-ICU environment were ventilated with a lung-protective strategy more frequently after an intervention reminding RTs to measure actual patient height and providing a tape measure to do so. A significantly higher percentage of patients had height measured rather than estimated after the intervention, allowing for more accurate determination of ideal body weight and calculation of lung-protective ventilation volumes. Measuring all mechanically ventilated patients’ height with a tape measure is an example of a simple, low-cost, scalable intervention in line with guidelines developed to improve the quality of care delivered to critically ill ED patients.

## BACKGROUND

Approximately 240,000 patients receive mechanical ventilation in US emergency departments (ED) every year.^1^ However, these patients frequently do not receive ventilation with a lung-protective strategy as outlined by recommendations from American and European critical care societies.^2^,^3^ An important element of a lung-protective ventilation strategy is low tidal volume ventilation.^4^ Since appropriate tidal volumes are based on predicted body weight by height, accurate assessment of patient height is crucial. In clinical practice, patient height is often estimated, although visual estimation of patient height by clinicians is imprecise and may lead to larger tidal volumes than would otherwise be indicated.^5^

Lung-protective ventilation strategies applied to patients at risk for acute respiratory distress syndrome (ARDS) may reduce the incidence of ventilator associated lung injury and ARDS.^6^–^8^ Previous investigations of ventilator strategies in the ED have demonstrated that a substantial percentage of patients are ventilated with a non-lung-protective ventilation strategy and have few adjustments made to the ventilator.^9^,^10^ Inappropriate ventilator settings with excessively large tidal volumes and increased airway pressures are injurious, even when administered for a relatively short period of time.^6^,^11^ This time becomes more and more important as critically ill patients board in the ED for longer periods. A recent multicenter, retrospective study showed that patients who receive lung-protective ventilation in the ED have decreased incidence of ARDS and decreased risk of death compared to patients who do not.^12^ However, in this study only 58.4% of patients actually received lung-protective ventilation in the ED. In addition, therapeutic interventions started in the ED are often carried forward during the patient’s stay in the intensive care unit (ICU),^13^ further highlighting the importance of starting lung-protective ventilation early.

ED-based intervention bundles to improve adherence to lung-protective strategies can improve patient outcomes.^14^,^15^ The Society of Critical Care Medicine/American College of Emergency Physician joint ED-Critical Care Medicine Boarding Task Force identified obtaining an accurate height to provide appropriately protective tidal volumes as a key component of mechanical ventilation practice in the ED, which is a simple but vital intervention to improve patient care.^16^ The objective of our study was to assess whether providing a tape measure to respiratory therapists, along with a best practice advisory (BPA) to measure patient height, is associated with improved compliance with patient height measurement and lung-protective ventilation. We hypothesized that encouraging measurement of patient height in the ED and provision of a tape measure would improve compliance with a lung-protective ventilation strategy.

## METHODS

### Design

This was a single-center, retrospective cohort study, designed to evaluate the results of a quality improvement initiative. The institutional review board at the University of Michigan reviewed and approved this study. This study is presented in accordance with the Strengthening the Reporting of Observational Studies in Epidemiology (STROBE) statement.^16^

**Population Health Research Capsule**

What do we already know about this issue?

Lung-protective ventilation is often not achieved in the emergency department (ED). Assessing patient height is crucial, and clinicians are often inaccurate in visually estimating patient height.

What was the research question?

Does provision of a tape measure impact lung-protective ventilation in the ED?

What was the major finding of the study?

Patients were more likely to receive lung-protective ventilation in the ED when a tape measure was provided.

How does this improve population health?

Providing a tape measure in the ED is a simple, low-cost intervention to improve lung-protective ventilation.

### Setting

The Michigan Medicine adult ED is part of a large academic medical center with approximately 75,000 ED visits per year. The ED-based ICU – the Emergency Critical Care Center (EC3) – is a hybrid ED-ICU setting.^18^

### Patients

This study included all adult patients with ventilator management performed in EC3 from February 16, 2015–November 3, 2019, which determined the sample size.

### Interventions

**Figure 1:**
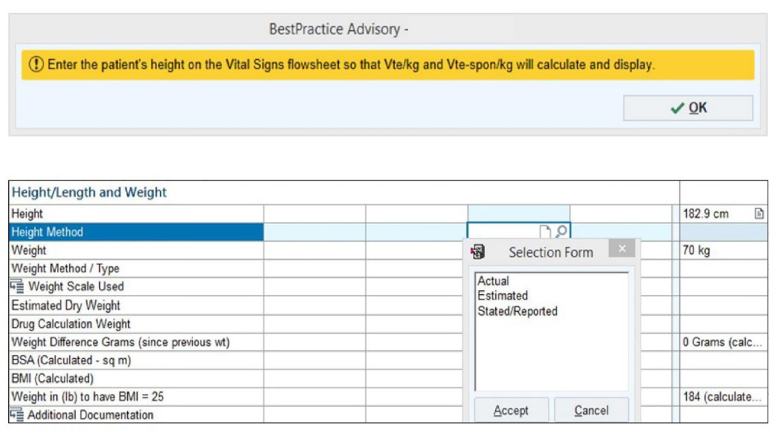
Best practice advisory requesting respiratory therapists to enter patient height and indicate how it was obtained. *wt*, weight; *sq m*, squared meter; *lb*, pound; *BMI*, body mass index.

On January 14, 2019, a ventilator-associated pneumonia (VAP) prevention bundle was instituted in EC3 for mechanically ventilated patients. As part of this bundle, respiratory therapists (RT) were provided with tape measures to accurately measure patient height. At the same time, a BPA was built into the electronic health record (EHR) reminding RTs to obtain patient height and to record whether the patient’s height was measured, estimated, or stated (Figure 1).

RTs performing clinical care were not aware of the ongoing study. We collected data on patient height and tidal volumes before and after the intervention. The pre-intervention period was from February 16, 2015, (EC3 opening date) through January 14, 2019 (the VAP prevention bundle initiation date). The post-intervention period was January 15, 2019–November 3, 2019.

### Statistical Analysis

The study sample was characterized with descriptive statistics and frequency distributions. We compared categorical variables from pre- to post- intervention using chi-squared tests. Continuous variables were compared from pre- to post-intervention using independent sample t-tests. We compared categorical variables from pre- to post-intervention using chi-squared tests and bivariate logistic regression analysis. We used multivariable logistic regression analysis to test for intervention as a predictor of measured height, statistically controlling for potential confounders (age, gender, and EC3 length of stay). Data were analyzed using IBM Statistical Package for the Social Sciences (SPSS) for Windows, version 26 (IBM Corp., Armonk, NY).

## RESULTS

We identified 54,188 tidal volume measurements in 2023 patients. We excluded from analysis the records of 197 patients with incomplete or missing data regarding tidal volumes or patient height. Our final analysis included 51,404 tidal volume measurements in 1826 patients over the study period, with a median of 21 measurements per patient (range 1 to 64 measurements per patient). The average variance in tidal volumes per patient was 12 milliliters (mL). Tidal volumes for each patient were averaged over the course of their ED-ICU stay and this average was used to determine whether lung-protective ventilation was achieved. In the pre-intervention period 1,579 (86.5%) patients were seen and 247 (13.5%) were seen in the post-intervention period. The sample was 43% female with no significant gender difference pre- and post-intervention (see Table 1).

Similarly, results from a bivariate logistic regression analysis showed that the odds of patient height being measured were 10 times higher post-intervention compared to pre-intervention (odds ratio [OR] 10.0, 95% confidence interval [CI], 6.7, 15.0). To rule out potential confounders, we conducted a multivariable logistic regression analysis of intervention as a predictor of measured height. Results showed that even when age, gender, and EC3 LOS were statistically controlled, the effects of the intervention on the odds of patient height being measured remained strong and significant (adjusted OR 9.9, 95% CI, 6.6, 15.0).

Although baseline compliance with a low tidal volume strategy was high, we found that more patients in the post-intervention group were ventilated with mean tidal volumes less than 8 cubic centimeters per kilogram (cc/kg) (84.5% vs 93.9%, P < 0.05). The difference in mean tidal volumes < 6 cc/kg was not significant (14.8% vs 17.0% P = 0.39). After the intervention, patients were ventilated with tidal volumes closer to 6 cc/kg ideal body weight compared to prior. The difference between delivered tidal volumes and 6 cc/kg of predicted body weight was less post-intervention (63±43 cc vs 36±76 cc, P < 0.05).

To address the potential confounding factor of increased awareness of lung-protective ventilation over time, we re-analyzed the data including only patients in the year prior to our intervention. We observed a similar increase in patients ventilated with tidal volumes less than 8 cc/kg after the intervention (89.2% vs 94.0%, *P* = 0.05) (Table 2).

## DISCUSSION

Providing RTs with a tape measure to measure actual patient height and creating a BPA in the EHR was associated with more frequent use of a lung-protective tidal volume strategy in an ED-ICU environment. Our study highlights the potential of a simple, inexpensive intervention to improve patient care.

Previous studies have found that clinicians are inaccurate when visually estimating patient height. Height measurements are biased toward the mean, which can result in significant overestimation of height in shorter stature patients.^5^ After our intervention, a significantly higher percentage of patients had height measured rather than reported or estimated, allowing for more accurate determination of ideal body weight and calculation of lung-protective ventilation volumes. Measuring all mechanically ventilated patients’ height with a tape measure is an example of a simple, low-cost, scalable intervention in line with guidelines developed to improve the quality of care delivered to critically ill ED patients.

We observed tidal volumes closer to the ideal 6 cc/kg predicted body weight after the intervention, even though no portion of our intervention required respiratory therapists to alter tidal volumes. It thus appears that a simple reminder to measure height also improved compliance with lung-protective ventilation. We did note that, although our baseline compliance with < 8cc/kg was high (84.5%), significantly more patients were ventilated with volumes < 8 cc/kg ideal body weight after the intervention (93.9%). This represents a substantial improvement in adherence to lung-protective ventilation in the ED.

The simplicity of the intervention allows it to be generalizable to any ED setting. Providing tape measures is a low-cost intervention that can help patients even in limited resource settings. In our ED-ICU environment, we observed a large and clinically meaningful increase in the measurement of patient height. Future studies could focus on the difference between estimated and measured height, the magnitude of the resulting difference in tidal volumes, and whether this impacts patient outcomes. Other important parts of a lung-protective ventilation strategy, including maintaining plateau pressure < 30 centimeters water and using optimal positive end-expiratory pressure, should also be evaluated.

## LIMITATIONS

We did not collect data regarding clinical indications for intubation, which may have impacted the tidal volumes used. We did not make additional height measurements to determine whether measurements by RTs were done accurately. In this study, we were unable to comment on other aspects of lung-protective ventilation such as prevention of barotrauma or atelectrauma. We also could not comment on the impact of improved compliance with lung-protective ventilation on clinical outcomes in this study; however, this was demonstrated in prior studies.

This was a single-center study in a hybrid ED-ICU environment, which may limit generalizability. We did not correlate compliance with lung-protective ventilation to specific physicians or respiratory therapists; it is unclear what impact individual practice patterns may have had on these results, including whether completing a critical care fellowship impacted this practice.

## CONCLUSION

Patients in an ED-ICU environment were ventilated with a lung-protective strategy more frequently after a simple quality improvement intervention reminding respiratory therapists to measure actual patient height and providing a tape measure to do so. Measuring all mechanically ventilated patients’ height with a tape measure is an example of a simple, low-cost, scalable intervention in line with guidelines developed by thoracic and critical care professional societies to improve the quality of care delivered to critically ill ED patients.

## Figures and Tables

**Table 1:** Pre- and post-intervention group characteristics.

	Pre-intervention n = 1579	Post-intervention n = 247	*P*
Female (%)	676 (42.8%)	107 (43.3%)	0.891
Mean age (years)	57.9 ± 17.6	60.4 ± 18.2	< 0.05
Mean height (inches)	67.3 ± 4.4	67.3 ± 4.2	0.939
Mean tidal volume (mL)	451.8 ± 86.4	425.3 ± 72.6	< 0.05
EC3 LOS (hours)	11.0 ±7.9	10.0 ± 6.7	< 0.05

*mL*, milliliters; *EC3 LOS*, emergency critical care center length of stay.

**Table 2:** Pre- and post-intervention measurement and tidal volume outcomes.

	Pre-Intervention (n = 1579)	Post-Intervention (n = 247)	*P*
Height used by clinicians to generate tidal volume			
Measured	51 (3.2%)	62 (25.1%)	< 0.05
Estimated	455 (28.8%)	57 (23.0%)	0.37
Stated	787 (49.8%)	94 (38.1%)	< 0.05
Not recorded	286 (18.2%)	34 (13.8%)	0.53
Mean tidal volume < 6 cc/kg	234 (14.8%)	42 (17.0%)	0.39
Mean tidal volume < 8 cc/kg	1332 (84.5%)	232 (93.9%)	< 0.05

*cc*, cubic centimeter; *kg*, kilogram.
